# Advancements in Soft Robotics: A Comprehensive Review on Actuation Methods, Materials, and Applications

**DOI:** 10.3390/polym16081087

**Published:** 2024-04-12

**Authors:** Yanmei Wang, Yanen Wang, Ray Tahir Mushtaq, Qinghua Wei

**Affiliations:** Industry Engineering Department, School of Mechanical Engineering, Northwestern Polytechnical University, Xi’an 710072, China; tahirmushtaqray@mail.nwpu.edu.cn (R.T.M.); weiqinghua@nwpu.edu.cn (Q.W.)

**Keywords:** material, actuating methods, application

## Abstract

The flexibility and adaptability of soft robots enable them to perform various tasks in changing environments, such as flower picking, fruit harvesting, in vivo targeted treatment, and information feedback. However, these fulfilled functions are discrepant, based on the varied working environments, driving methods, and materials. To further understand the working principle and research emphasis of soft robots, this paper summarized the current research status of soft robots from the aspects of actuating methods (e.g., humidity, temperature, PH, electricity, pressure, magnetic field, light, biological, and hybrid drive), materials (like hydrogels, shape-memory materials, and other flexible materials) and application areas (camouflage, medical devices, electrical equipment, and grippers, etc.). Finally, we provided some opinions on the technical difficulties and challenges of soft robots to comprehensively comprehend soft robots, lucubrate their applications, and improve the quality of our lives.

## 1. Introduction

Robots are in the service of humans and are used to handle complex and tedious work (e.g., repetitive and precise lifting or placing of objects, providing real-time information and accurate feedback), usually composed of rigid materials such as metals and alloys [1]. With the development of science and technology, even industrial robot control has made outstanding achievements in computer vision, deep learning, imitation learning, and other fields. However, due to the diversity of working environments, there is still a need to continually improve and optimize robot control operation in terms of reliability, flexibility, adaptability, and friendly interaction with users [2,3]. Furthermore, for fragile or soft targets and harsh operating environments, applications are limited due to their rigidity and limited adaptability. Owing to their outstanding flexibility and adaptation, research on soft robots has burgeoned, which is also the main distinguishing factor from traditional robots made of rigid materials.

Commonly, soft robots comprising materials with moduli close to those of soft organisms (e.g., fat, cartilage, and skin), ranging from 10^4^ up to 10^9^ Pa [4,5], such as gels, functional polymers, and other flexible materials. Generally, soft robots have remarkable properties, such as safe human/robot interaction [6,7] and multiple degrees of freedom [8,9]. Consequently, soft robots possess great promise for biomedical applications [10], bionic movements (like grasps [11], crawls [12], swims [13], jumps [14], etc.), camouflage [15], and soft electronic devices [16,17]. Moreover, soft robots can be applied in hazardous and unstable environments, exploration in confined spaces, locomotion on uneven terrain, and toxic environments encountered after natural disasters and collapsed buildings [18].

Although soft robots have achieved some success, especially in biomedical applications, soft grippers, and soft electrical devices, there are still several challenges. Compared to traditional rigid robots, which can choose many common parts directly, soft robots have few generalized components [8]. In addition, due to the specificity of the structure and motion of soft robots, higher requirements are placed on the actuators in terms of flexibility, pliability, deformation capability, and energy consumption [19]. In the meantime, even though some reviews about soft robots exist, they either focus on the used materials (e.g., hydrogel [6,20]), emphasize the driving method (such as magnetic actuation [4,21]), or conduct analysis based on the fabrication process [22,23]. Therefore, this paper will review soft robots through driving methods, used materials, and applications which are missing in previous review studies.

The novelty of this review displays the existing soft robot actuation methods (ten kinds), available materials (SMM, elastomer, and fibers, etc.) and their characteristics (based on modulus), and the common application areas (e.g., medical care, flexible electrical devices, multimodal locomotion modes, camouflage, etc.), and comprehensively investigates their features, advantages, and disadvantages. Therefore, it provides a reference for subsequent soft robot application research to improve the function of the soft robot and better serve human beings.

## 2. Actuating Method

The actuating methods of soft robots are closely related to the selection of materials, the design of the structure, the motion modes, and the application area. Commonly, the actuating approaches contain humidity, temperature, electricity, magnetism, pH, pressure, light, cable/tendon, biotic, etc. The following will list their detailed working principles.

### 2.1. Humidity Actuating Soft Robots

**Figure 1:**
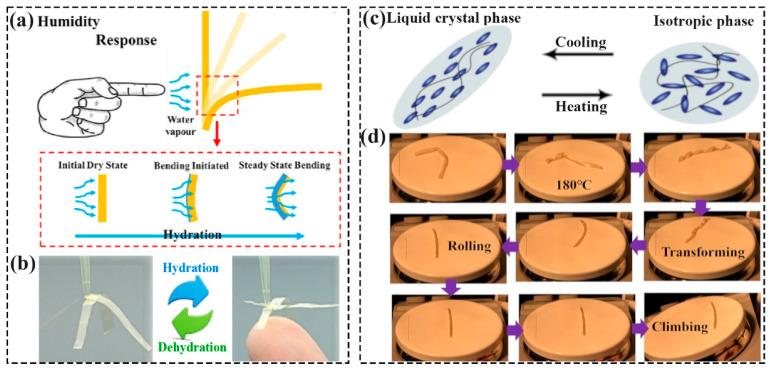
(**a**) 1. Schematic illustration of humidity-driven deformation; Reprinted with permission from the study presented in [25], License Number: 5760600153293, Copyright 2024 Elsevier. (**b**) The reversible motion of the gripper; Reprinted with permission from the study presented in [25], License Number: 5760600153293, Copyright 2024 Elsevier. (**c**) Schematic illustration of LCEs [27]. (**d**) Shape-changing and movement processes of the specimens. Reprinted with permission from the study presented in [29]; License Number: 5760561077746, Copyright 2021 Elsevier.

In nature, most botanical movements are due to fluid transportation (generally water) in and out of the plant tissue, like the opening and closing of pine cones, ascribed to the hygro-expansive properties of plant cells and the volumetric change in response to moisture content [24]. In light of this, Shin et al. [24] reported a bilayer ratchets structure robot based on a hygroscopically responsive film made of aligned nanofibers. The utilized material could rapidly expand and contract in the longitudinal direction according to the changes in humidity, leading to the imbalance deformation of the robot structure and ultimately driving the motion. As shown in Figure 1b, Li et al. [25] have fabricated a reversible humidity-triggered gripper based on the cellulose nanofiber films according to the hydration/dehydration process. Odent et al. [26] 3D-printed (SLA) a multi-armed gripper, which can rapidly expand from an initially flat to bent state, and close within 10 min in stained blue water.

### 2.2. Thermal Actuating Soft Robots

According to the source of heat energy, thermally driven soft robots commonly contain direct heating (heat conduction) and indirect heating (photothermal, Joule heating, which will be described in detail in the electrical and photothermal actuating section). The used materials mainly include liquid crystal elastomers (LCEs) and shape memory materials. For example, LCEs possess both liquid crystal (self-organizing) polymeric elastomer (entropic elasticity) properties, thus, can reversibly change their shape after applying external stimuli, like thermal energy (Figure 1c) [27]. Therefore, Roach et al. [28] designed and fabricated the soft and long LCE fibers using the DIW method and knitted (loom weaving and sewing) fibers into complex textiles. Upon heating, textiles could change shape by their breathable pores based on variations in temperature, such as the wearer’s increased body or environmental temperature. Zhai et al. [29] fabricated an LCE-based soft robot that could complete the untethered rolling under the high-temperature incentive. As displayed in Figure 1d, when the temperature was above 160 °C, the prepared specimens deformed into tubules and began to move spontaneously. By changing the size and curvature direction of the robots, the velocity and rolling direction of the samples can be adjusted. Moreover, the research of soft robots based on their thermal responsive shape memory polymer/shape memory polymer composite (SMP/SMPC) was also attractive. For example, Chen et al. [30] prepared a tetherless soft swimming robot that could be pre-programmed, and the directional propulsion was without power and auxiliary electronic equipment. Therein, the surrounding temperature controlled large displacements of bistable elements (the shape memory effect of the muscles) to realize the motion of robots.

### 2.3. pH Actuating Soft Robots

**Figure 2:**
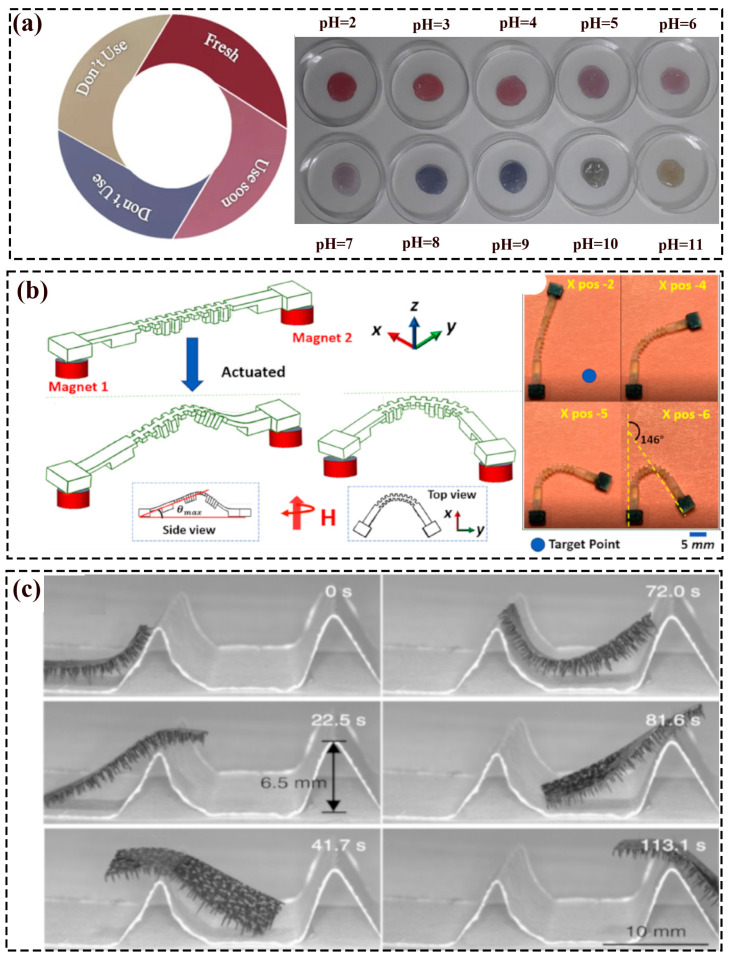
pH and magnetic driving soft robots: (**a**) The pH indicator and the color response of nanocellulose at varied pH; Reprinted with permission from the study presented in [33]; License Number: 5760680196760, Copyright 2019 Elsevier. (**b**) Schematic and the actual movement pictures of the untethered compliant soft robot; Reprinted with permission from the study presented in [34]; License Number: 5760650166841, Copyright 2020 Elsevier. (**c**) The robot locomotion in varied situations [35].

The movement of pH-driven soft robots is due to the principle of anisotropic expansion, and the structure is generally composed of two or more layers with different pH-trigger deformation capabilities [31]. For instance, Duan et al. [31] have fabricated bio-hydrogel actuators that consist of a chitosan layer and C/CMC layer, which could fulfill the rapid, reversible, and repeated self-rolling movements, gripping and deforming into varied shapes under pH-actuation. Han et al. [32] proposed a pH-responsive drug delivery system, which released drugs by the stimulus of the pH value (alkaline/acidic atmospheres). In addition, the hydrogel bilayer could achieve various designable movements through added parallel elastic fringe arrays on the side of the structure. As depicted by Figure 2a, Moradi et al. [33] proposed a pH stimulus indicator that could change color according to the pH values to monitor the freshness of the fish.

### 2.4. Magnetic Actuating Soft Robots

**Figure 3:**
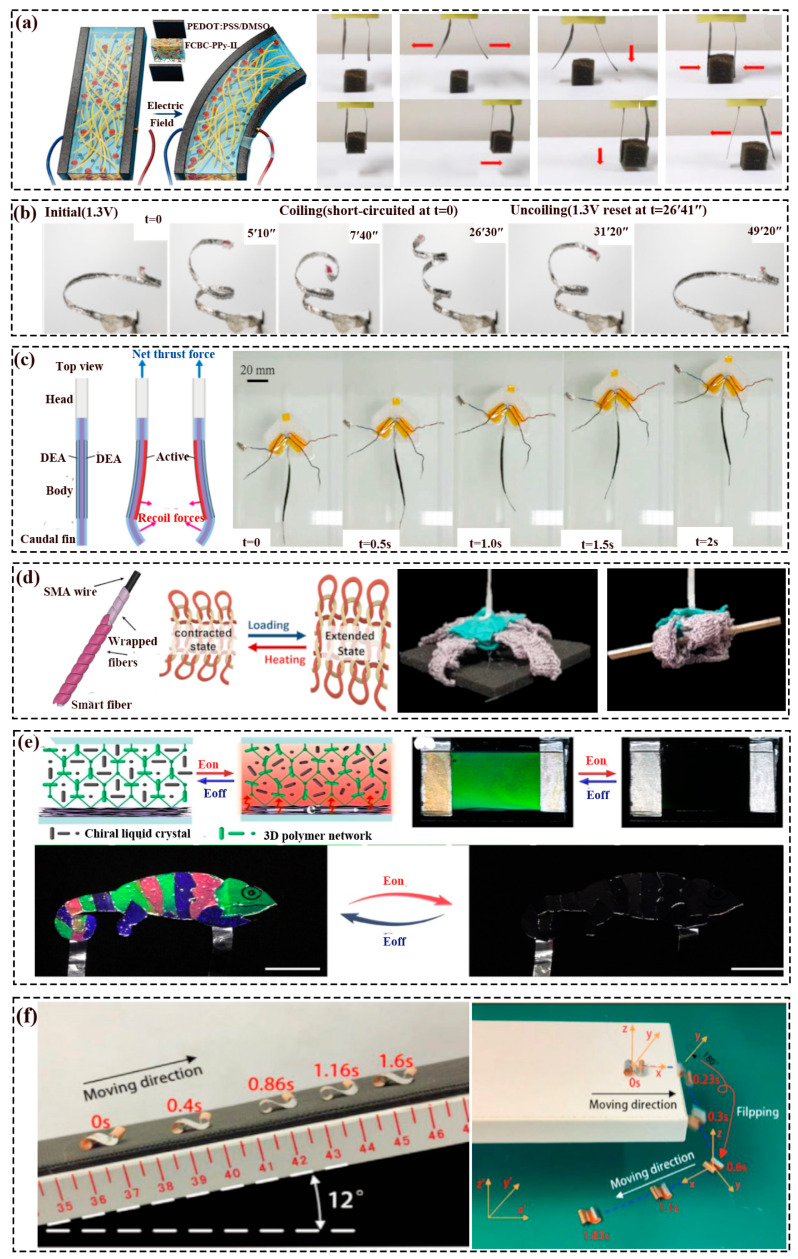
Electrically actuating soft robots: (**a**) Schematic diagram of the electrically induced ion migration soft robots and the gripper working process (the red arrows were the moving direction); Reprinted with permission from the study presented in [42]; License Number: 5760690026167, Copyright 2020 Wiley-VCH GmbH. (**b**) The working figure of the tendril soft robot [44]. (**c**) Schematic of the fish working and the swimming process under 5 kV [47]. (**d**) Schematic of the active fiber forming process, knitted fabric working principle, and the gripper grasping objects [48]. (**e**) Schematic of electrochromic principle and the disguise process by applied DC voltage; Reprinted with permission from the study presented in [49], License Number: 5760770707891, Copyright 2022 Wiley-VCH GmbH. (**f**) BFFSPR climbs a 12° slope and moves after falling and flipping from a high platform [50].

Under the action of the magnetic field, the magnetic material in the soft robot with the magnetization curve of a variable size and direction will deform to align with the magnetic field (such as bending, elongation, and contraction), finally causing the movement of the structure. As one of the untethered external actuations, the magnetic actuating soft robots can circumvent the limitation of external power sources and additional wires (e.g., the electrically and pneumatic driven), and can even be operated in different mediums such as vacuum, air, and liquids [34]. Therefore, Pan et al. [34] proposed an untethered compliant soft robot with multi-modal locomotion for on-demand drug delivery applications, shown in Figure 3b. The robot consisted of a body and anterior and posterior legs with embedded magnetic materials, and had three degrees of freedom. Additionally, the robot could store drugs inside the reservoir and release them after it reached the goal, and the load weight could reach thirteen times itself. Ji et al. [36] 3D-printed (DLP) multiple magnetic driving soft actuators (e.g., gripper) via magnetic photosensitive resins with the ability to bend, deform, transport cargo, etc. Zhang et al. [37] fabricated (DIW) magnetically driven soft robots (e.g., an inchworm soft robot and gecko soft robot based on the magnetic slurry and Ecoflex materials. By the attraction of rubidium magnets on the back, inchworms and geckos could crawl on different slopes. Wang et al. [38] have reported a sea anemone-inspired soft robot that consisted of tentacle-like magnetoelectric sensors and a magneto-stimulated shrinkable hollow body to protect itself from being swept away. The robot could sense the speed of water to turn on/off the external magnetic field, correspondingly controlling the shrinkage/recovery of its body. Kim et al. [39] presented a soft continuum robot that could perform omnidirectional steering and navigate via the magnetic field. Lu et al. [35] presented an untethered magnetic soft millirobot with many tapered feet structures with an ultrafast moving velocity, loading more than its 100 times weight, and a brilliant obstacle-crossing capability, depicted in Figure 3c. Sitti et al. [40] proposed a soft robot that possessed the ability to grab and transport cargo via rolling movement under the exterior rotating magnetic fields.

### 2.5. Electrically Actuating Soft Robots

According to the mechanism of electrically actuating soft robots, which can be divided into electrically induced ion migration (electrochemical), the electroactive polymers elastomer changing sizes or shapes by Joule heating or electrochromic- and piezoelectrical-driven responses are detailed as follows.

#### 2.5.1. Electrically Induced Ion Migration Soft Robots

One of the representative mechanisms of the electrical-triggered actuator is composed of one ionically conductive electrolyte sandwiched by two electrically conductive electrodes, and the redistribution of cations and anions under an applied voltage led to the bending of the structure [41,42]. As shown in (Figure 3a), Wang et al. [42] have prepared a gripper that could complete grasping and moving tasks based on the FCBC-PPy-IL membrane. Herein, the motion of the gripper was ascribed to that under the electrical stimulus (square input of 0.5 V/1 V at 0.1 Hz.), the cation (EMIM^+)^ and anion (BF4^−^) movement inside the electrical membrane, leading to the relative volume discrepancy of two sides, in the macroscopic, was bending to the anode. Han et al. [43] 3D-printed (DLP) electroactive hydrogel (EAH) soft robots that can grip (or transport) an object and have bidirectional locomotion of the human-like EAH structure in an electric field. As shown in Figure 3b, Must et al. [44] have displayed a tendril-like soft robot, which could realize reversible stiffening and actuation by low voltages (1.3 V) based on the electrosorption of ions on the electrodes.

#### 2.5.2. Dielectric Elastomer-Based Soft Robots

Based on the working principle (changing sizes or shapes in response to an electrical stimulus) of the voltage actuation of dielectric elastomer (DE). Cheng et al. [45] presented a tactile force sensor by the DE, which displayed varied force according to the changed capacitive of the structure and also possessed enhanced and tunable sensitivity. Cao et al. [46] have reported a soft robot made of DE and paper-based feet with a velocity of 0.02 body length/s, which was actuated by the alternated expansion/contraction of its deformable body and adhesion/detachment of its feet. As presented in Figure 3c, Jun et al. [47] proposed a soft bionic fish by DE, which swims according to the periodic deform of the body and caudal fin. Herein, the maximum velocity was up to 37.2 mm/s, which was about 0.25 times of the body.

#### 2.5.3. Joule Heating-Actuated Soft Robots

Strictly speaking, Joule heating-actuated soft robots belong to the thermal-driven type, which converts electric energy into heat to drive the robot to work (like changing shape) indirectly, and the most commonly used material is a shape memory alloy (SMA). For instance, Gong et al. [51] have designed a bionic soft tongue, which could fetch solids or liquids. Therein, the contraction or the curling of the tongue depended on the layout of the SMA, e.g., vertical or horizontal. It took about 2 s for the tongue tip to curl to grip objects under the currents of 0.57 A, and the currents of 0.73 A contracted upward using about 1 s. Park et al. [52] proposed a suit-type wearable robot (STWR), which used an SMA-based fabric muscle (SFM) as the actuator. The step response experiments of the SFM that lifted barbells to the marked location found out that, compared to the 2 kg target, the 4 kg target-consumed time was strikingly slower. Shin et al. [48] presented a soft gripper made of the active fibers (Figure 3e, wrapped the conventional fiber on the SMA) and inactive fibers that could lift varied objects (e.g., cotton, a cup, chopsticks, etc.) without damage. Liu et al. [53] proposed a soft gripper with three fingers based on the SMA, which possessed variable stiffness that could grasp compliantly at a low stiffness and clutch forcefully at a high stiffness.

#### 2.5.4. Electrochromic Soft Robots

In nature, some creatures can rapidly change their color, texture, or posture reversibly to adapt to the situation, like chameleons, octopus vulgaris, and cuttlefish. Therefore, the soft robot that could achieve color-changing under electrical stimulus burgeoned. For example, Yun et al. [54] fabricated an all-transparent stretchable electrochromic supercapacitor device. Ling et al. [55] reported air-working electrochromic artificial muscles (EAMs) capable of displaying deformation (elongation and contraction) by changing color. Herein, the EAMs generated a contractile stroke of approximately 12% during stable operation in the air, exhibited multiple color changes (yellow–green–grey) under ±4 V voltage actuation, and the reflectance contrast reached as high as 51%. Zhang et al. [49] have proposed a soft electrochromic camouflage structure, which could reversibly switch bright color states and deep black states when voltage was applied, as seen in Figure 3e.

#### 2.5.5. Piezoelectrical Soft Robots

Vibration is a fast motion often observed in nature (like honeybees) and our daily lives (e.g., strings). Therefore, soft robots worked by vibration have attracted the attention of researchers due to their outstanding response time and output forces [50]. For example, Chen et al. [50] reported a bionic piezoelectrical soft robot made of inverse piezoelectric-actuated PVDF film, Ag electrodes, and a passive layer film(Cu). As displayed in Figure 3f, the movement utilized the resonance of a double-helical shaped structure. The maximum moving speed reached 42.8 body lengths per second (BL/s), and the average turning velocity was 482° s^−1^ at the first resonance frequency when the applied voltage was 200 Vpp. Wu et al. [56] presented a fast-moving and ultra-robust soft robot based on a curved unimorph piezoelectrical structure that consisted of a PVDF layer, Pd/Au electrodes, adhesive silicone, and a PET substrate. The soft robot worked based on the large vibration amplitude and the bouncing gait mechanism to generate a wavy motion near its resonant frequency. Its moving velocity reached 20 BL/s when the AC actuated and moved, even though the loaded weight was 1 million times heavier than itself.

### 2.6. Cable/Tendon Actuating Soft Robots

**Figure 4:**
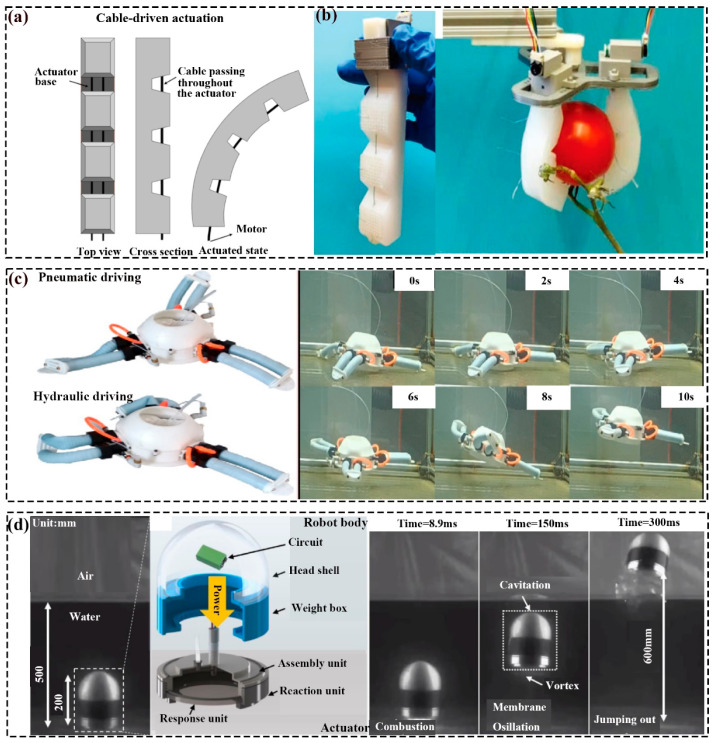
Cable/tendon and pressure driving soft robots: (**a**) Working principle of cable-driven actuation [21]. (**b**) Photograph of the as-fabricated actuator and the graph of picking off tomatoes from its stem; Reprinted with permission from the study presented in [57]; License Number: 5760791352048, 2020 WILEY-VCH Verlag GmbH and Co. KGaA, Weinheim. (**c**) The pneumatic/hydraulic-actuated states and the swimming and crawling process; Reprinted with permission from the study presented in [67]; License Number: 5760810820767, 2023 Elsevier B.V. All rights reserved. (**d**) Schematic of the structure and working process of underwater jumper; Reprinted with permission from the study presented in [70]; License Number: 5761041451817, 2020 Elsevier Ltd. All rights reserved.

As shown in Figure 4a, the cable/tendon-actuated soft robot uses a cable embedded in the soft construction with one end fixed at a specific position and controlled by the other end to contract/expand to actuate the movement of the structure [21]. Based on this mechanism, Chen et al. [57] have described a cable-driven soft finger, which possessed the ability to move quickly, bend perception, and self-supply, and perform basic hand movements, such as grasping a tomato (Figure 4b). Lee et al. [58] prepared soft actuator-utilized free-sliding SMA wires as tendons, and the maximum weight of the targets was 1.5 kg, as seen in Figure 4c. George et al. [59] have studied an SMA-driven continuum robot, which could bend in different spatial directions by activating one or two wires simultaneously.

### 2.7. Pressure-Driving Soft Robots

Pressure-driving soft robots commonly utilize internal inflatable cavity (e.g., different channels or chamber structures) deformation due to the uneven stress under the pressure stimulus, then complete the spatiotemporal movement. Generally, there are three kinds: pneumatic, hydraulic, and combustion pressure. The following is the detailed information.

#### 2.7.1. Pneumatic Actuating Soft Robots

Pneumatic actuating soft robots fill the gas into the soft structure through varied airways, leading to the uneven pressure of the structure to achieve different motion trajectories. Inspired by earthworms, Ge et al. [60] have presented a pneumatically driven soft robot that could move in horizontal and inclined platforms. Herein, the motion modes could be adjusted through the robot and the supporting platform frictional coefficient under controlled inflation/deflation arrangements. In the same way, Ariel et al. [61] proposed a type of pneumatical soft robot that could move in a pipe. Liang et al. [62] presented the microscale soft pneumatic actuators (SPA) with varied working patterns ascribed to the diverse structures (e.g., the discrepancy in shape and dimension). Huang et al. [63] presented a pneumatic soft robot that could adjust its construction according to the structure and dimension of the targets through expansion or contraction. The experiments verified its maximum load and diameter were 2.5 kg and 310 mm, respectively.

#### 2.7.2. Hydraulic Actuating Soft Robot

Compared to the pneumatical actuators, the hydraulically responsive soft robots work under hydraulic pressure (commonly water) [20,64]. For example, Xie et al. [65] designed a hydraulic soft actuator with three hydraulic chambers that could complete spatial movement like extending, bending, and steering. Chen et al. [13] presented three underwater soft actuators, namely water hydraulic soft grippers and a water hydraulic soft biomimetic fishtail. Robert et al. [66] presented an untethered soft robotic fish with the ability to record the daily life of the following aquatic organism. Herein, the velocity and turn could be controlled by the tail propulsion frequency and the deflection under the varied hydraulic pressure. Combining the characteristics of air pressure and hydraulic pressure, Chen et al. [67] have prepared a hybrid-actuated (pneumatic/hydraulic) soft robot that could swim and crawl in the water atmosphere. As presented in Figure 4c, switching the type of pressure could shift the movement pattern of the robot. That is, the high-pressure water actuated the crawling motion, and the high-pressure gas controlled the up-and-down movement (swimming).

#### 2.7.3. Combustion Actuating Soft Robot

In addition to the pneumatic, hydraulic, and hybrid-driven methods, explosion-motivated soft robots are also used. Therein, the combustible gas in a soft structure was ignited by a specific energy that caused an explosion and rapidly generated high pressure, leading the robot to move. For instance, Zhou et al. [68] explored a chameleon-like soft robot that fulfilled the long-distance extension rapidly, which worked according to the liquid C4H10 explosion pressure. Aubin et al. [69] proposed a soft robot that possessed the ability to crawl with a high velocity and accomplish large vertical leaping based on methane combustion. The maximum jumping speed was 16.9 cm/s, and the crawling velocity was nearly 16 cm/s. Yang et al. [70] have proposed an underwater jumper that was actuated by propane combustion and instantly jumped out of the water. The jump height was up to 6 times its size in 300 ms, i.e., the maximum distance was 1.2 m (Figure 4c).

### 2.8. Light Actuating Soft Robots

According to the working mechanism of light-actuated soft robots, they can be divided into photothermal and photochemical types [71]. Generally, the working principle of the former is similar to thermo-sensitive SMM, wherein light energy is converted into heat energy and indirectly heats the structure to realize shape-memory movement. As for photochemical soft robots, the used materials usually possess photochemical groups (e.g., azo-benzene, anthracene, cinnamon, and coumarone). The light illumination with specific wavelengths can change the chemical groups into a new state by chemical crosslinking, and macroscopic manifestation is the motion of the soft robots.

#### 2.8.1. Photothermal Soft Robots

**Figure 5:**
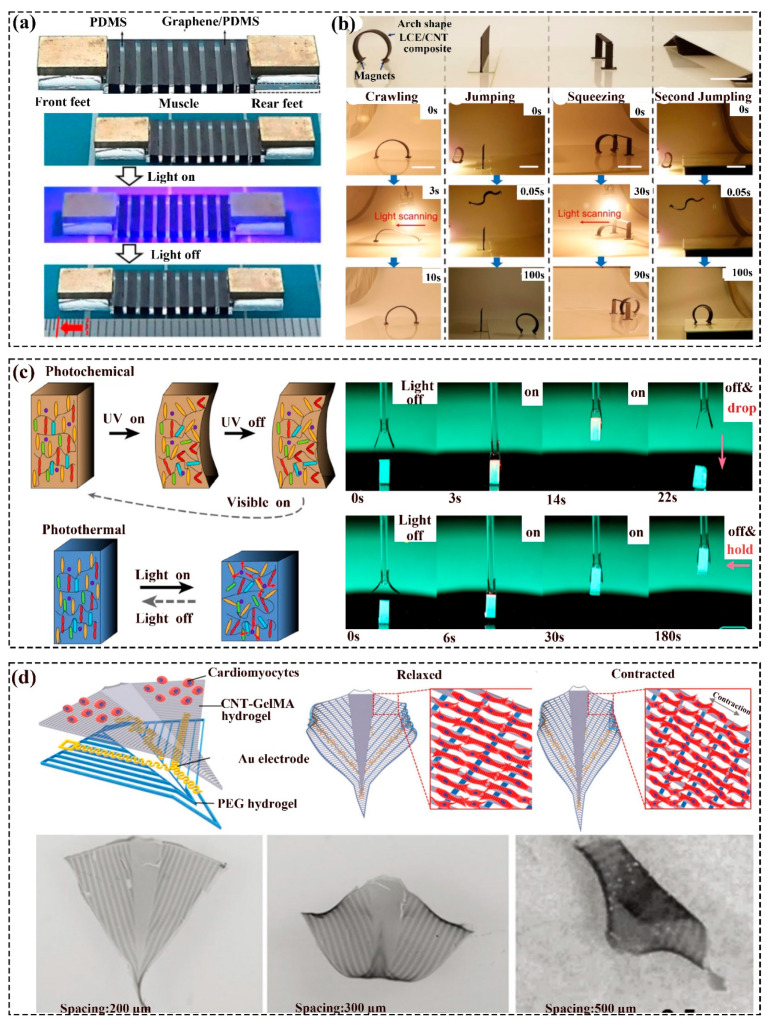
Light and bio-actuating soft robots: (**a**) The structure of the Geca-Robot and its snapshots under ten on/off cycles of UV light irradiation; Reprinted with permission from the study presented in [72], License Number:5760840556829, 2020 Elsevier Ltd. All rights reserved. (**b**) Multimodal locomotion (crawling, jumping, and squeezing) of the soft robot powered by light; Reprinted with permission from the study presented in [12], License Number: 5760850206277, 2019 WILEY-VCH Verlag GmbH and Co. KGaA, Weinheim. (**c**) Schematic of the photochemical/photothermal incentive; the original working process (grip, deliver, and release) under the red-light irradiation and the gripper worked in the grip–deliver–hold procedure after synergistic photoactivation [75]. (**d**) Schematic illustration of the robot structure and the rolling snapshot of the robot under different scales; Reprinted with permission from the study presented in [77], License Number: 5761091340636, 2018 WILEY-VCH Verlag GmbH and Co. KGaA, Weinheim.

The photothermal soft robots commonly contain photothermal particles and other flexible materials. The robot converts the light energy into thermal energy, which in turn drives the shape of the material to change and finally achieve movement. Wang et al. [72] have reported a footed soft robot (Geca-Robot) based on the photothermal property of graphene and the elasticity of PDM, which had good terrain adaptation and load-bearing ability. As displayed by Figure 5a, being illuminated with wavelengths ranging from ultraviolet (UV) to infrared (IR), Geca-Robot unidirectionally traveled with a caterpillar-like gait on terrains of varying roughness, slope, and dryness and carried loads weighing approximately 50 times its mass. Zhan et al. [73] fabricated a photothermal-responsive hydrogel soft robot capable of fulfilling the reversible motion within a few seconds by regulating the intensity and the IR illumination route. Furthermore, due to the properties of the uniaxially aligned mesogens in a monodomain, they become disordered as the temperature grows, leading to the reversible shrinkage of the LCE. Cai et al. [12] demonstrated a soft robot consisting of LCE/CNT that could complete movements like crawling, squeezing, and jumping by light illumination (Figure 5b). Herein, under light irradiation, the maximum jumping height and length were about 8 and 5 times of the own body. Sun et al. [74] prepared a peristaltic crawling soft robot based on the photothermal TiNS/AuNP hydrogel. Herein, the hydrogel could complete periodic elongation and contraction under the turn on/off function of the IR and possess anisotropy endowed by the magnetic alignment of cofacially oriented TiNS along the cylindrical axis.

#### 2.8.2. Photochemical Soft Robots

The photochemical soft robots commonly contained photosensitive groups, such as azobenzene, which had cis to trans isomerism and the two isomers that could be transformed reversibly under UV and VIS illumination. For example, Markus et al. [75] have proposed a soft reconfigurable gripper based on the collaboration of photochemical and photothermal effects that were made of azobenzene-based liquid crystal polymer networks(LCN). As displayed in Figure 5c, the gripper worked in the process of the grip–lift–drop through the switch of a red light (photothermal effect to deform). In the meantime, if activated by UV first, the gripper was reprogrammed and could hold the objects after turning off the red light. Liu et al. [76] have presented an inverse opal actuator (inchworm walker, logic electric circuit, and engine axis) that consisted of the monodomain azobenzene polymer layer and the polydomain azobenzene inverse opal structure. Herein, the polymer side worked reversibly based on the photochemical effect (trans/cis isomerization of azobenzene mesogens), and the other side worked according to the photothermal actuation.

### 2.9. Bio-Actuating Soft Robots

Referring to the abilities of biological systems to sense, process, and respond to their surroundings in real time, bio-actuating soft robots, capable of adapting their responses to dynamic environments, have occurred accordingly. As revealed by Figure 5c, Shin et al. [77] developed a bioinspired dual-layer soft robotics system made of a PEG hydrogel base layer, a GelMA layer embedded with CNTs, and seeded cardiomyocytes. The robot could be actuated by electrical force, motivating the movement of cardiomyocytes through the Au microelectrodes located under the cell layer to imitate the stingray motion. Justus et al. [78] have prepared a biosensing soft gripper that could sense environmental information based on engineered bacteria and light-emitting diode (LED) circuits. The three-finger gripper could sort the objects into the designed location according to the detected chemical information.

### 2.10. Hybrid Actuating Soft Robots

Although classical, one-way-driven soft robots are widely available, and they still have drawbacks like reconfigurability, cyclability, and application limitation [79]. Therefore, soft robots with multiple driving synergies have attracted the attention of researchers. For instance, Liu et al. [79] have presented a bifunctional soft robot with magnetic/light-actuated based on SMP and Fe microparticles. Herein, under the IR (860 nm) illumination and permanent magnet triggered the scroll that could complete the open–lock–closed–open again cycle, as well as the grabber that could fetch and lay down the blueberries/cherry tomatoes with no damage, and the weight of the objects could hold up to 20 times of itself. Yuya et al. [80] proposed a biohybrid robot that packaged the skeletal muscle tissue in a collagen construction to ensure humid environments even in the air. Therein, the electrical stimuli could cause the robot to bend and push a bead to move due to the contractions. Based on a PPC/Fe_3_O_4_/SMP nanocomposite, Gu et al. [81] 4D-printed the IR/magnetic dual-stimulated soft robots for hazardous chemical operations. Herein, the gripper reached the designed site due to the folded part shape recovered through the magnetic triggering. Following, the gripper released the drugs by the NIR illumination.

### 2.11. Summary of the Actuating Methods of Soft Robots

In conclusion, as shown in Table 1, every kind of actuation for soft robots has advantages and disadvantages [18,41,82]. The humidity-driven soft robots possess the properties of reversible, wireless, high sensitivity, and recyclable small output force and low deformation precision. Thermally driven soft robots are widely used with low costs, untether, and are safer than UV and electric drives. In addition, since there is a large number of available materials, such as the LCE, with reversible thermal deformation properties, SMP has tunable properties through the change fabrication method. However, it has the disadvantage of a lower deforming accuracy and poor real-time deformation. The pH-driven types are reversible and wireless and usually have low precision and require multiple layers of materials with different properties to complete the varied motion modes. The electrically driven ion migration type has a low voltage, a high energy conversion rate, and a small output force. The DE-driven type has high energy density, high strain, a high strength ratio, and relatively high control precision while requiring high voltage applications, leading to the leakage of current and easy electrical breakdown. Joule-heat controlled soft robots have low noise, low voltage, high distortion, smooth motion, low control accuracy, and hysteresis. The electrochromic soft robots can provide real-time deformation through color change, high adaptability, and precision, but the material is limited. The piezoelectric triggering type has a high output force, large working bandwidth, and high voltage. A magnetic drive robot is remotely actuated and tetherless, has a fast response time, a low deformation accuracy, and is difficult to control. In addition, the need for external equipment to assist the robot in working would limit its dimensions. The cable/tendon actuation through the traditional motor drive possesses a short response time, high control accuracy, and energy loss due to friction. Pressure-driven types require rigid auxiliary equipment and have the risk of leakage. Herein, pneumatic actuation is the most widely used with a wide range of gas sources, is easy to control, lightweight, and frictionless, but has a low load capacity. In the meantime, hydraulic drives have a higher drive force and load capacity than pneumatic types and faster travel speeds. The explosive-actuated soft robot works using instantaneous high pressure with large strokes and a short response time. However, it is necessary to reuse the appropriate explosive material every time due to its low service life, low precision, and the robot being hard to control in its moving direction.

The photothermal-actuated soft robot can adjust its performance according to the demand (such as SMP), and the deformation is unidirectionally irreversible, the precision is low, and the output force is small. The photochemical-actuated motion is usually reversible, with a longer response time and a small output force. The NIR can penetrate the tissue to perform remote control, but UV is harmful to humans. The bio-driven type has good biocompatibility, a low deformation accuracy, and a low output force. Hybrid soft robots combine the advantages of the used drive modes with the capability of cyclic, reconfigurable, and multifunctional characteristics, which need more auxiliary equipment and complex motion control. Given the variety of drive methods for soft robots, the materials used and the application areas also vary. Therefore, the optimal actuating method should be selected based on the requirements and material characteristics during the practical application research.

## 3. Materials of the Soft Robots

In conjunction with soft robotics actuation methods, commonly used materials include hydrogels, shape memory materials (SMMs, such as SMP, shape memory hydrogel (SMH), and SMA), and flexible materials, e.g., PDMS, silicone, thermoplastic polyurethanes (TPUs), elastomers, fibers and fabrics. Depending on the properties of utilized materials, the fabricating process of soft robots is different; correspondingly commonly covering casting [83,84], molding [64], pressing [85], spin coating [62,86], laminating [87], electrospinning [24,54], 3D-printing (such as fused deposition modeling (FDM) [88,89], direct ink writing (DIW) [29,90], stereolithography (SLA) [73,91], selective laser sintering (SLS) [92], poly jet technology [93], etc.) or the combination of them, which allows for the design of the sophisticated structure. The following section will describe the actuation methods and types of soft robots prepared based on the characteristics of the different materials.

### 3.1. Hydrogels

Hydrogels are made of water and hydrophilic homopolymers or copolymer chains arranged in a network structure, and the crosslinked polymer chains render them into a three-dimensional elastic solid [20]. Therein, the crosslinked polymer networks are capable of absorbing or desorbing large amounts of solvent in response to the environmental conditions (for example, temperature [84,91], light [82,94], electrical [43,84], magnetic [39,95], hydraulic [64,96] or pH [31,32]) to realize the macroscopic deformation of the materials. Hydrogels have good transparency(~99%), and the performances of the hydrogels (e.g., stretchability, toughness, fluidity and softness) can be easily controlled via covalent or ionic bonds during the synthesis procedure [97,98]. Furthermore, by modifying the network structures and functional groups of hydrogels or adding conducting fillers or ionic salts into the hydrogels, hydrogels can gain electrical, ionic conductivity, electrochemical, and simulated biological functions [99,100,101]. Combined to the drive mode, the hydrogel-based soft robot research mainly covers actuators [102], sensors [13,103,104], communicators [94,105,106], etc., detailed information can be seen in Table 2. Moreover, even though hydrogel possesses outstanding features such as low elastic modulus (585 Pa–100 kPa) [20,107,108] and good flexibility, it cannot work in high temperatures and strength environments due to the large volume fraction of water that is easy to dehydrate when exposed to the air environment, which in turn limits its application range.

### 3.2. SMMs

SMMs are materials that can recover to their initial status (such as shape, color, transparency) from a temporary state under external stimulation (e.g., thermal [89,125], water [126], electrical, magnetic [127], PH [32], light [128]). Generally, SMMs contains SMP [125] and its composites (SMPC), SMAs, shape-memory ceramics (SMCs) and shape-memory hydrogels (SMHs) [129]. Up to now, as displayed by Table 3, the SMM-based soft robot application research covers multi-gait soft robots [130,131], electrical devices [127], grippers [89,132,133], sensors [134,135,136], etc.

#### 3.2.1. SMP and Their Composites

SMPs are elastic polymer networks composed of netpoints and appropriate stimuli-sensitive molecular switches. Herein, the net points in charge of the shape of the polymer network can be chemical (covalent bonds) or physical (intermolecular interactions) crosslinked amorphous or crystalline copolymers, while molecular switches respond to external stimuli [71]. The modulus of SMP range from ~MPa to ~GPa [137,138]. The SMP possesses the features of high elastic deformation (the strain of majority materials above 200%), low production cost, tailored actuating temperatures, tunable stiffness, are easy to process, possess biocompatibility, biodegradability, and fast shape response rates (in the scale of seconds to minutes depending on actuation methods and the shape deformation mechanism [127]). Moreover, to overcome the deficiencies of SMP, the fillers or fibers (e.g., carbon-based particles, inorganic/organic ingredients, dye, etc.) are combined with the SMP to form a multiphase SMPC to enhance the properties of pure SMP [133]. Therefore, the SMP and their composites-based soft robots research also gained the attention of scholars. For instance, Wang et al. [89] fabricated a soft gripper using 3D-printed(FDM) thermo-responsive SMPC (TPU/PCL blends). Lv et al. [139] proposed a PVA/MSCNT porous composite with multi-responsive (thermal, NIR light, water) shape memory actuating and self-powered sensing functions (triboelectric nanogenerator). Zhang et al. [133] explored a soft gripper through the SMPC material, which covered a structure design, preparation, and application performance study. Zhang et al. [140] proposed an electrically driven, fast-response, adjustable stiffness soft gripper using a 3D-printed hybrid multi-material, which could lift an object weighted between 10 g–1.5 kg.

#### 3.2.2. SMAs

The shape-memory effect of SMAs stems from the crystallization transformation of martensitic and austenitic phases corresponding to the temperature changes. Herein, the SMA possesses a high response stress (~hundreds of MPa), an elastic strain of 5%, high elastic modulus (span from ~10 GPa to ~100 GPa according to the temperature), and good thermal conductivity (commonly electrically actuated) [133]. For instance, the displacement of SMA can be controlled through Joule heating with a relatively low voltage power due to the SMA contracts when electricity is applied [53]. Moreover, SMA has uniform deformation and low noise. Thus, SMA-based soft robots have been widely studied. For example, set the SMA as a tendon (similar to the muscle–tendon structure of humans) and combine with soft materials (e.g., silicone, PDMS, TangoBlackPlus, SMP) to fabricate the soft robots through the control of the SMA length (e.g., prosthetic hand, rehabilitation soft robots [141], grippers [53,58,142,143,144,145,146], crawling soft robots [147], fish-like underwater robot [130]). Moreover, according to the electrical resistance changes during the actuation of SMA, they also can be applied as sensors [148].

#### 3.2.3. SMH

Once the stimulus is applied, the SMH also recovers to the original state from the temporary shape acquired through stretching, compression, or folding [149]. Due to their sensing ability and flexibility, SMH-based soft robots are also attractive. For example, Song et al. [150] 4D-printed biodegradable SMH models that could quickly and sensitively perceive subtle external touches. Guo et al. [134] designed a strain sensor to detect and differentiate handwriting samples, Morse codes, and human movements, ascribing to the shape memory hydrogel which converted the strain change of the actuator into a specific electric signal under external motivation. Zhang et al. [151] fabricated a two-layer actuator with PDN gels that is self-healing, and the shape-memory performance is endowed by dehydration and water absorption. Huang et al. [135] proposed a stretching sensor with controllable thermal-response shape memory properties by the ionic conducting hydrogel.

**Table 3:** Shape memory materials-based soft robots.

Materials	Working Mechanism	The Form of Soft Robots	Reference
SMP/SMPC	Thermal actuation	Gripper	[89,132,133,152]
	Magnetic actuation	Gripper	[127]
	Electrically actuation (Joule heating)	Gripper	[140]
	IR light/magnetic actuation	Gripper Scroll	[79]
	Thermal/IR light/water actuation	Pressure and humidity sensors/triboelectric nanogenerator	[139]
	IR light/magnetic actuation	Soft robot	[81]
SMA	Magnetic actuation and n	Gripper/Soft robot	[147]
	Electrically actuation (Joule heating)	Gripper/fish/Fast moving soft robot/Flame-retardant soft robotics/A suit-type wearable robot/Soft wearable exoskeleton/actuator/Tri-legged soft bot	[12,52,53,58,130,141,142,143,145,147,148,153,154]
SMH	Thermal actuation	Actuators	[155,156]
	Thermal actuation	Pressure sensor	[150]
	IR light actuation	Microrobots	[73]
	The different swelling ratios of the two layers	Actuator	[151]
	Change the electric signal according to the varied strain	Strain sensor	[134]
	Thermal actuation	Stretching sensor	[135]

### 3.3. Flexible Materials

In addition to hydrogels and SMM, the commonly flexible materials applied in soft robots are silicones and their polymers, elastomers, fibers, and so on. As revealed in Table 4, their application mainly covers artificial limbs, multi-gait soft robots, grippers, generators, sensors, etc., and the following sections will describe the properties of each material and the corresponding type of soft robots.

#### 3.3.1. Silicones and Their Polymers

Silicone, an elastomer (rubber-like material) containing silicon, carbon, hydrogen, and oxygen, with a modulus around 0.4–5 MPa [152,157,158], can be applied in automotive, electronic, and medical devices, etc. As a kind of silicone-based polymer, the modulus of PDMS is about 1–3 MPa [159] and was widely used (e.g., in biomedical devices, flexible electronics, e-skin, and self-healing apparatus). Ascribing to their properties (such as biocompatibility, high stability, and good mechanical properties, etc.) [159,160], silicones and their polymers are used to fabricate soft robots that can be used alone or in collaboration with other materials to enhance the performance of soft robots with desired characteristics. Up to now, soft robots mainly contain grippers [13,161], other kinds of actuators [62,65,88,162], and sensors [163].

#### 3.3.2. Elastomers

Thermal polyurethane is a kind of elastomer composed of an amorphous soft segment and crystalline hard segment, commonly formed through chemical synthesis based on short/long-chain diols and diisocyanate [89], and the modulus is between 0.36–64.31 MPa [164,165,166]. The dielectric elastomer (DE), belonging to the electroactive polymer, is a kind of soft material (modulus from 0.17 MPa to 3 MPa [167,168], which can change dimensions or shapes under an electrical incentive. Meanwhile, LCE changes its molecular order parameters by temperature triggers (directly heating [169,170] or photo warming indirectly [171]) and then realizes the movement of elongation, contraction, bending and twisting [172]. Herein, its modulus ranged from 0.1 MPa to 1 GPa based on the temperature, alignment, and crystallization [173,174]. Therefore, scholars are researching elastomer-based soft robots and have already achieved outstanding results, for instance, pneumatically actuated soft actuators [175,176,177], rolling soft robot [19], bionic muscle [9], submersible robot [178], sensors [179], and soft prosthetic hands [92,180], etc.

#### 3.3.3. Fibers

Fiber is a one-dimensional material with a large aspect ratio that can be fabricated as two/three-dimensional fabrics via twine/knit/weave techniques. Attributed to the varied matrix material, the modulus of the fibers ranges from ~KPa to ~MPa (e.g., Geniomer is 0.48 MPa, nylon fiber is 0.69 Mpa, poly(styrene-b-(ethylene-co-butylene)-b-styrene) fiber is 1.56 MPa, LCE fiber is 2 MPa, and polycarbonate fiber is 789 MPa) [28,181,182]. Due to its unique characteristics, e.g., pliability, lightweight, flexibility, various levels of porosity up to 99%, and suitability for wearable systems, the research on flexible fiber or fabric-based structures for soft robots has attracted much attention. For example, Wang et al. [183] have presented reversible soft actuators (rainy curtain, breathable fabric, and crane), actuated according to the environmental water content by the gel-state alginate fiber. Guo et al. [184] prepared stretchable optical temperature sensors using thermal-sensitive upconversion nanoparticles and polymer-based optical fibers that could monitor the real-time temperature of the targets. Wu et al. [185] prepared a temperature/pressure sensor with the silk fiber and carbon nanotubes(CNT)/ionic liquid. Liu et al. [186] fabricated the textile-based pressure-sensor arrays by Ni-coating on the CNT fabric. Qi et al. [28] fabricated a series of reversible textiles(bicep muscle and shirt) based on the LCE fiber.

#### 3.3.4. Others

Furthermore, other flexible materials are used to fabricate soft robots. For example, Drotman et al. [187] 3D-printed (Polyjet technology) a pneumatic quadruped soft robot through TangoBlackPlus/VeroClear materials, which could move on uneven terrain. Zatopa et al. [93] fabricated a soft hydraulic-controlled octopus-like robot by 3D-printing the mixture prepared by Tango+ and Vero White. Ge et al. [188] 3D-printed a micro soft pneumatic gripper via the Tangoplus FLX930. Hisham et al. [189] have prepared soft and monolithic pneumatic fingers through 3D-printing (FDM) thermoplastic elastomers (TPEs). Zhai et al. [29] developed a liquid crystal elastomer-based rolling soft robot. Wang et al. [190] presented an actuator with 3D-printed (FDM) conductive PLA on the copy paper, which was activated through joule heating. Heung et al. [191] 3D-printed (ACEO^®®^-Wacker Chemie) a pneumatic soft hand for rehabilitation.

**Table 4:** Soft robots based on flexible materials.

Materials	Working Mechanism	The Form of Soft Robots	Reference
Silicones and their polymers	Pneumatic actuation	Actuator	[13,62,88,192,193,194,195]
	Measure the resistance caused by pressure	Pressure sensor	[163]
	Hydraulic actuation	Actuator	[65]
	Magnetic actuation	Actuator	[196,197]
	Thermal actuation	Actuator	[162]
	Electrically actuation (Joule heating)	Gripper	[8,161]
Elastomers	Pneumatic actuation	Actuator/Bionic hand	[92,175]
	Hydraulic actuation	Grippers	[198]
	Cable actuation	Soft prosthetic hand	[180]
	Electrical actuation	Rolling soft robot/Gripper/Submersible robotic/Mimetic muscle	[19,177,178]
	Change the resistance according to the strain	Strain sensor	[179]
	Change the capacitive according to the external force	Force sensor	[45]
Fibers	Converting the moisture level into voltage output	Textile sensor	[199]
	Under NIR excitation, the UCNPs generated thermal-sensitive dual-wavelength emissions, enabling ratiometric readout temperature	Temperature sensor	[184]
	Convert thermal change to stable output power	Temperature sensors	[200]
	Change the resistive according to the Temperature/Change the capacitive according to the force	Temperature/pressure sensor	[185]
	Change the resistive according to the varied vibrations and forces pressure	Pressure sensor	[186]
	Humidity actuation	Self-locomotive ratcheted actuator/rainy curtain	[24,183]
	Thermal actuation	Smart Textiles/Artificial Muscles	[28]
Other flexible materials	Piezoelectric effect under an AC driving voltage to change the shape	Soft robot	[56]
	Electrically actuation (Joule heating)	Actuator	[190]
	Electrosorption of ions on flexible electrodes by low voltages (1.3 V)	Tendril-like soft robot	[44]
	Pneumatic actuation	Gripper/Legged robot/Soft hand exoskeleton	[176,187,188,189,191,201]
	Hydraulic actuation	Actuator	[93]
	Magnetic actuation	Drug delivery/Inchworm/Gecko Soft Robot/Gripper	[34,36,37]
	Thermal actuation	Self-propelling soft robot	[29]

### 3.4. Summary of the Materials Used in Soft Actuators

According to the properties of each material and its type of soft robot, we can find out that, due to the low modulus of hydrogels and good flexibility, there are more studies on the soft robot application of sensors. Meantime, SMP/SMPC could be actuated by diverse methods, and most of the applications were actuators due to their adjustable performance. The SMA-based soft robot should cooperate with soft materials to complete the specified task ascribed to the low strain and high strength of SMA which was commonly was Joule thermal-actuated. Generally, the SMH-based soft robot application environment has low force requirements and ambient temperatures. Most of the fibers-based soft robots were sensors. As for other flexible materials, most of them were newly prepared composite materials with a wide application range. Therefore, in the study of soft robots, the use of materials should be a comprehensive analysis of the practical requirements and application situation to research the most suitable and valuable materials.

## 4. Application

### 4.1. Camouflage

**Figure 6:**
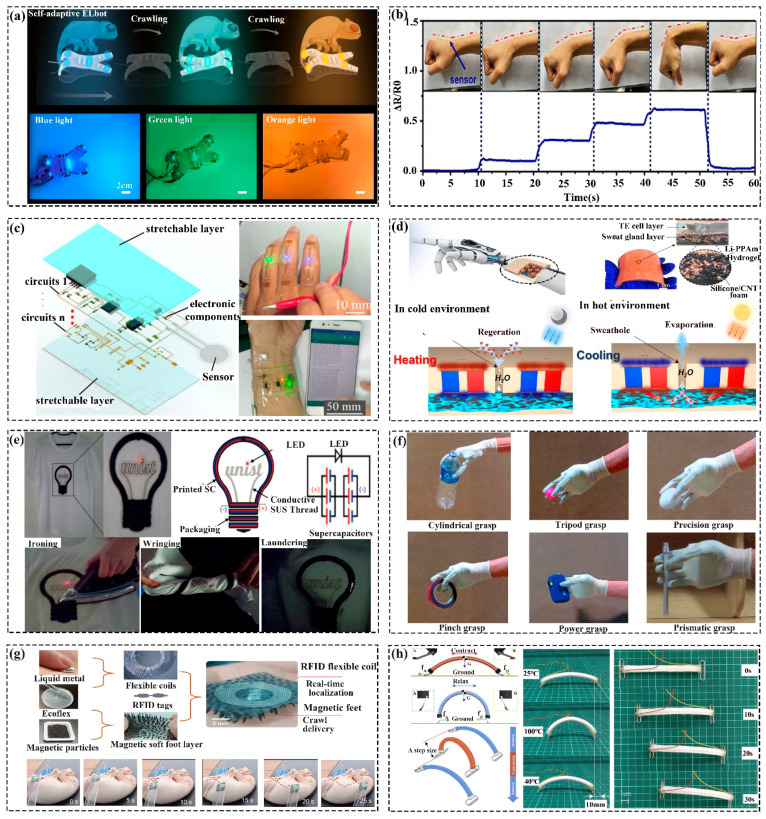
Application of the soft robots: (**a**) Schematic illustrating the working process and the real-time color-changing of the ELbot [15]. (**b**) The signal from the soft sensor that was attached to the wrist at different angles; Reprinted with permission from the study presented in [203], License Number: 5761821174596, 2022 Elsevier Ltd. All rights reserved. (**c**) The exploded graphic of a stretchable circuit system, the LED stretchable circuit on the fingers and the WPMP attached to the wrist of a human; Reprinted with permission from the study presented in [207], License Number: 5761840026438, 2021 Elsevier B.V. All rights reserved. (**d**) The schematic of the thermo-e-skin and its working principle; Reprinted with permission from the study presented in [208], License Number:5761840618790, 2023 Elsevier Ltd. All rights reserved. (**e**) Schematic of the white T-shirt with SC/SS wire that lighted up the LED and its working mode (laundering, wringing, and ironing); Reprinted with permission from the study presented in [121]. License Number: 5761240911146, 2018 WILEY-VCH Verlag GmbH and Co. KGaA, Weinheim. (**f**) The bionic hand grasped varied targets; Reprinted with permission from the study presented in [215], License Number: 5761841217826, 2017 Elsevier B.V. All rights reserved. (**g**) The multi-legged robots and the real-time moving position under magnetic field actuation in the pig stomach; Reprinted with permission from the study presented in [221], License Number: 5761850219976, 2023 The Author(s). Published by Elsevier Inc. (**h**) The working principle of the robot and the moving process (the temperature from 40 °C to 100 °C with a period of 25 s and 60–100 °C with a period of 5 s, respectively), the A and B were the name of the two feet; Reprinted with permission from the study presented in [225], License Number: 5761851010876, 2019 Elsevier B.V. All rights reserved.

Animals actively change their coloration or display dynamic body patterns for camouflage, protection, and warning. This has enlightened the camouflage research of soft robots that change color under set stimuli. For instance, Lee et al. [94] reported that electrochromic devices could change purple to block specific light spectra and disguise them, while voltage is applied to the electrochromic devices. Combined with the soft quadrupedal robot (fabricated by DIW technology) and the sensing units, Zhang et al. [13] reported a soft robot that could instantly change its color to fit the atmosphere for disguise, details as seen in Figure 6a. Kim et al. [202] prepared the adaptive artificial camouflage robot by collaborating with the AgNW/TLC-based bionic skin, color sensors, and feedback control equipment. Therein, the chameleon-like robot could change its color in real time after acquiring the color of the environment.

### 4.2. Electronic Devices

Based on the diverse working principles and applications, the current research on soft robot applications as electronic devices mainly includes sensors, flexible circuits, electronic skin, energy storage, and power generation devices, etc. The following chapter will show detailed examples of these devices.

#### 4.2.1. Soft Sensors

Soft robots that gather information (e.g., the changing of volume, thickness, humidity, chemicals, force, and capacitance) from the external environment and transform them into acceptable data, like color and electrical signal, are sensors. According to the working mechanism, soft sensors generally contain color, strain, capacitance, resistive, magnetic, temperature, chemical sensors, etc. For instance, Hwang et al. [203] prepared a self-adhesive biocompatible soft strain sensor that could monitor the motion of human beings and the biosignals based on the combination of the self-adhesive polyurethane (SAPU) and the silver nanowires (Ag NWs). Herein, as shown in Figure 6b, the deformation (strain) of the objects could be detected by the changing resistance. Qin et al. [103] reported a simple and universal adaptive color platform (or color sensor) ascribing to the external triggers the changed the dimension (thickness) of the soft hydrogel film, which could be displayed through the visual color alteration in real-time. Liu et al. [104] presented a bio-sensor composed of hydrogel/elastomer and multiform genetically engineered bacterial cells. The application research of living patches and biosensing gloves proved the responsive ability to multiple chemical factors. Liu et al. [112] fabricated a wearable strain sensor that could detect biological motions (e.g., joint movement, breathing, and blood pulsation) based on the dynamic change of the CNCs−Fe^3+^ coordination bonds. Wu et al. [204] demonstrated a tactile sensor that could convert the external force into inductance capacitance (LC) and output it as readable digital-frequency signals. Zhao et al. [205] developed a soft sensor made of ring-shaped origami magnetic films, which could detect the mechanical movement of the targets.

#### 4.2.2. Soft Circuits

Flexible soft circuits are electrical devices that can continue to work after undergoing different motions (e.g., bending, twisting, stretching, etc.). For instance, Wei et al. [206] fabricated a flexible and stretchable circuit based on the AgNWs/PDMS composite that achieved stable conductivity in large tensile strains (0–50%) and demonstrated some wearable applications. As shown in Figure 6c, Lin et al. [207] explored stretchable circuit boards (SCBs), fabricated by the combination of stretchable wires/circuits, shaped polyimide-based substrate layers, sensors, and other auxiliary devices. Based on the principles of electrical signals instantly revealing the changed strain, experiments confirmed that patching the SCBs on the finger could light an LED and the wrapped wireless pressure monitoring prototype (WPMP) on the wrist could continually work for 48 h when the tester performed regular activities (e.g., dining, typing on computers, and writing).

#### 4.2.3. Electronic Skin

Inspired by the functions of the skin (e.g., regulating body temperature, protection, sensing, and metabolism), flexible electronic skins (e-skins) that mimic the workings of skins have attracted the attention of researchers. For example, Zhang et al. [208] investigated a thermo-e-skin capable of regulating body temperature composed of Li-PAAm hydrogel, silicone/CNT foam, the encapsulation elastomer, and related flexible electronic devices. The skin could adjust its temperature situated at 35 °C based on the principle that when the ambient temperature (10–40 °C) was higher than the set value, the layer of electrical equipment in the e-skin cooled down, Li-PAAm hydrogel evaporated to decrease its temperature as well. Similarly, when the temperature was low, the electric heating device layer warmed up, and the hydrogel in the skin absorbed moisture from the environment, as displayed in Figure 6d. Ge et al. [209] fabricated the difunctional e-skins that possessed the capability to detect the approach and touch motions simultaneously based on the principle that the approach could incur the modification of the environmental magnetic field, and the touching force triggered the resistance change. Based on the same principle, Majidi et al. [210] introduced flexible tactile e-skins made of silicone elastomer and magnetic microparticles that could sense the imposed contact and force. Yan et al. [211] presented soft tactile e-skins capable of percept the varied magnetic flux densities incurred by the external normal/shear force.

#### 4.2.4. Soft Power Sources

Due to portable and flexible electric devices becoming more prevalent in our everyday lives, there is a rising requirement for lightweight, pliable, and efficient soft power sources. Commonly, they contain energy storage devices and energy generators [20]. For example, supercapacitors (SCs) are a new type of energy storage device between traditional capacitors and rechargeable batteries which are environmentally friendly, with a high specific power and long service life. Li et al. [120] synthesized the all-gel-state fibrous SCs based on the GO/PANI/hydrogels composites that could power two tandem LEDs in flat or curved situations. Lee et al. [121] demonstrated a new class of wearable SCs (consisted of AC/MWCNTs/[EMIM] [TFSI], [EMIM] [TFSI]/UV cross-linkable thiol-ene monomer/SiO_2_ nanoparticles, PDMS/curing agent/MWCNT, and the electroconductive SS wiresi) fabricated on the T-shirts, as displayed in Figure 6e, it still worked (lighted the LEDs) when the shirts was undergoing the treatment of launder, wring, ironing, and folding.

Furthermore, the research based on flexible structures to harvest external energy (e.g., mechanical energy, electrostatic, triboelectric, electromagnetic, etc.) and converting it into electrical energy has yielded some promising results. For instance, Zhou et al. [124] reported a device that could translate mechanical energy (the change of stress) as electricity based on the principle of redeployment of the cations and anions of the hydrogel-based electrodes. Xu et al. [123] fabricated a novel hydrogel-based TENG capable of providing energy for the sensor based on movements like bending, twisting, and stretching. Liu et al. [212] fabricated a triboelectric soft robot (TESR) system which had a good adaptability for crawling on different substrates. The inchworm-like robot could crawl autonomously and perform simple tasks through the triboelectric effect, which consisted of a soft deformable body and two triboelectric-adhered feet. Sun et al. [213] proposed a soft robot (TENG-Bot) suitable for TENG powering and controlling based on uni-directional dielectric elastomer actuators (DEAs). The crawling speed of the TENG-Bot could be up to 2.2 times its body (110 mm/s), and the maximum load was 40 g. Li et al. [214] developed a magnetized microneedle-array (MA) flexible TEHG as a self-powered sensor for monitoring human motions based on the electromagnetism and triboelectrification of the microneedles. The TEHG could monitor the walking/jogging paces and the arm motion when set in the insole and inner elbow, respectively.

#### 4.2.5. Other Flexible Electronics

In addition to the soft electronic devices mentioned above, there exists research that possesses research value. Studies such as that performed by Yang et al. [105] demonstrated a fully organic liquid-crystal device (OLCD) made of the encapsulated liquid crystal DE cell and ionic conducting gels, which possessed a disguised ability according to the varied display form. Kim et al. [86] proposed a strategy for fabricating printable and highly stretchable conductors (stretchable electrodes and skin patches)) through the collaboration of Ag ink, the Ecoflex elastomer, hydrogel, and tape. Gao et al. [106] developed a novel photochromic hydrogel displayer that could change color under the illumination of UV for only 2 s, which could be applied in artificial intelligence, visual storage devices, and color displayers, etc.

### 4.3. Medical Soft Robot

#### 4.3.1. Prosthetics and Reconstruction Robots

Amputation and hemiplegia in chronic stroke survivors causes limited mobility in their limbs, which severely affects their daily lives. Therefore, bionic prosthetics as an assistive medical device can improve the quality of life for those who have lost the mobility of their limbs. Udupa et al. [215] have prepared a soft asymmetric bellows pneumatic actuator using rubber, as revealed in Figure 6f. The band with muti-fingers was able to grasp objects of different shapes and sizes (e.g., bottle, egg, tape, ping-pong, pen, etc.). Moreover, its grasp/lease velocity was about 5–8 times its weight, and the weight of the whole arm was half that of conventional prostheses. Yan et al. [216] proposed a tendon-actuated soft prosthetic hand with five fingers by 3D-printed (DLP) nylon that could complete basic grasping movements and display the common gestures (e.g., V, OK, and the number 6 in Chinese sign language). Alireza et al. [180] presented a soft cable-triggered prosthetic hand (X-Limb) corresponding to practical requirements via 3D-printed (FDM) the TPU90. The X-Limb achieved a weight of 253 g, three grasp types (e.g., power grasp, pinch grasp and tripod grasp), the strength of grip was up to 21.5 N, the finger bending velocity was 1.3 s, and the durable test verified that it could work at least one year.

#### 4.3.2. Surgical and Drug Delivery Soft Robot

Minimally invasive surgery (MIS) is increasingly attractive for its advantages of minor wounds, less bleeding, lower complication rates, and quick recovery. Nevertheless, the rigid medical device for MIS interventional therapy still needs to be improved, which triggered soft surgical manipulation research and achieved some outstanding outcomes. For example, Wang et al. [217] presented a cable-actuated soft robot system used for pericardial space operations, which aims to lighten the burden on doctors and enhance operational precision. Hoshiar et al. [218] fabricated a flexible microrobot which used external magnetic field steering to improve the guidance. Kim et al. [219] developed a soft microrobot steered by the external magnetic field. Herein, the working angle of the robot was between 21.1°–132.7° under the magnetic field (15 mT intensity) controlling.

As for gastric ulcer symptoms, targeted therapy is an effective and preferable option due to the difference in the location and size of the lesion for varied patients [220]. In light of this, Ye et al. [221] have explored a remote-controlled (external magnetic field) soft multi-legged robot that could target delivery made of liquid metal, Ecoflex, and magnetic particles. As Figure 6g revealed, combined with the RFID tracking/localization technology and magnetic actuation, the robot has diverse moving directions and forms (such as curling, rolling, floating, diving, and crawling). In collaboration with the vision guide of the endoscope, Dong et al. [222] have fabricated a remotely active (magnetic field) soft robot made of magnetized NdFeB and two sides of double-sided tape for treating pig stomach ulcers. In the same way, Alexis et al. [223] prepared an ingestible patch and plug for gastric ulcer treatment, actuated by the magnetic field. Therein, when the robot reached the designed position, it could deploy immediately via hydration.

### 4.4. Other Soft Actuators

In addition to the above application research, according to the environments and requests, the commonly soft actuators are divided into gripper, legged, crawling, hopping, and swimming robots.

Therefore, grasping is the fundamental function of robots, and related research is also encouraging [224]. For example, Zheng et al. [82] presented a reversible gripper that could grasp, transport, and release cargo in air based on the hydrogel with a reverse thermal responsive bilayer composite structure. Wang et al. [84] fabricated a soft gripper through 3D-printing (FDM) thermo-responsive SMPC (TPU/PCL blends). Nie et al. [191] established a hydraulic (water) soft actuator (WHSA) fabricated directly by 3D-printing (FDM) TPU-95A material. The results showed that WHSA, without leakage after 8 h, continued working, and the maximum grasping diameter/weight was 120 mm/2 kg, respectively. Ze et al. [120] reported the inductive heating (magnetic inductive heating of low-coercivity particles) and actuation soft robotic grippers based on the M-SMPs, which could effectively be unlocked and locked for energy-efficient operations in its functions. Wang et al. [138] combined the SMA and SMP to fabricate a soft finger that could grasp variable stiffness targets. Shiblee et al. [148] fabricated an SMH-based bilayer gripper based on the diverse expansion rate under the synergistic functions of heating and water. Schönfeld et al. [125] designed a gripper by the PEU that could precisely grip, hold, and release an egg.

Tang et al. [225] prepared a soft crawling robot through silicone/paper clips/nickel wire/nylon fiber, which was actuated by Joule heating (5V voltage). As displayed in Figure 6h, the crawling motion of the robot was according to the synthetic action of structure deformation and the friction generated from the clip-based foot and the platform. Mihai et al. [226] fabricated a four-legged, multi-gait crawler via a multilayer fabrication method, and its moving direction and speed could be adjusted via the applied voltage. Yang et al. [227] presented a water-walking soft robot through the miniaturized gold nanorods, LCN, and superhydrophobic copper foams. Under the NIR illumination, the walking velocity could be up to 5.7 mm/s. Pan et al. [228] fabricated a frog-like jumping robot, actuated by the explosive(hydrogen/oxygen gas). Herein, the jumper could realize six consecutive jumps, the jumping height and distance up to 0.6 m and 1.2 m, respectively.

## 5. Conclusions and Outlook

In summary, this work has analyzed soft robots using actuation methods, commonly used materials, and application areas. We have found that hydrogel has a lower modulus and good biocompatibility. The driving method was mostly electric driving, and the application areas were flexible electronics, such as sensors, electronic skin, and supercapacitors, due to the easy adjustment of their properties of SMP and SMPC, varied drive modes, and large application areas. SMA is commonly driven by photothermal or Joule heating, and its applications are mostly grippers. The most used flexible materials were elastomers. For example, LCE can fulfill reversible deformation for gripper research under thermal actuation. Therefore, the above analysis can guide the subsequent in-depth research on soft robots according to practical needs.

However, although the current research on soft robots has achieved outstanding results, some shortcomings still need to be addressed. For example, hydrogel-based soft robots have poor mechanical strength due to high water content. For the SMP/SMPC-based soft robots, either with irreversible (one-way SME) characteristics that respond quickly or possess reversible (like photochemical) abilities consumed longer to actuate, SMA-soft robots have a limited deformation range and usually require an additional electrical device. Therefore, subsequent research can focus on the following aspects. (1) Combining the characteristics of each material (the flexibility of hydrogels, the autonomous deformation characteristics of shape memory materials, and the varied processing technologies of other flexible materials) to prepare high-performance multi-function soft robots. (2) Collaborating with photochemical and magnetically sensitive SMPC to further study the remote driving untethered reversible soft robots. (3) Integrating flexible energy generators, soft circuits, and sensors to realize autonomous energy supply, perception, and decision-making for thorough research of bionic robots, such as SMAs for bionic bone, SMPs as muscle, and hydrogels for e-skin. (4) Successful cooperation of soft robots with industrial robot control methods to further optimize the autonomous deep learning for motion planning, draws on the advantages of the soft robot and control modes to provide better convenient and safe services for human beings.

## Figures and Tables

**Table 1:** Advantages and disadvantages of different actuating soft robots.

Actuating Methods		Advantages	Disadvantages	Reference
Humidity		Reversible/Untether/High sensitivity/excellent cyclicity/Safe	Multi-layer structure/Small output force/Low precision	[24,25,26]
Thermal		Safe/Reversible LCE and some of SMM)/Low cost/Untether	Poor real-time property/Low precision	[27,28,29,30]
pH		Reversible/Untether	Double layer structure/Low precision	[31,32]
Electrically	Ion migration	Low voltage (from one to several volts)/High energy conversion efficiency	Small output force	[43,44]
	Dielectric elastomer	High strain (10–100%)/High energy density/High power-to-weight ratio	High voltage (1–10,000 V)/Unsafe/Easier electrical breakdown/Leakage current	[45,46]
	Joule heating	Low noise/low voltage/High distortion and smooth motion	Low precision/Hysteresis	[51,52,53]
	Electrochromic	Visual display in real-time/Adaptability/Good precision	Limited materials	[54,55]
	Piezoelectrical	High force/Large working bandwidth	Large voltage	[50,56]
Magnetic		Contactless/Fast response time	Low precision/Externally large devices/Difficult to control/Limited dimension	[34,35,36,37,38,39,40]
Cable and tendon		Short response time/Good precision	Energy loss due to friction	[21,57]
				[58,59]
Pressure	Pneumatic	Easy control/Fast working cycle/Lightweight/No friction	Leakage/Externally rigid control and power system/Low loads	[5,60,61,62,63]
	Hydraulic	High loads/High actuating force/High stability/High stroking velocity	Leakage/Externally rigid control and power apparatus/	[13,65,66,67]
	Explosive	Short response time/Large stroke	Limited lifespan/Uncontrolled direction/Low precision/Limited materials/Leakage	[59,68,69]
Light	Photothermal	Contactless/NIR can penetrate through biomaterials with low losses/Fast response time/Adjustable properties	Low precision/Small output force/Irreversible	[12,72,73,74]
	Photochemical	Contactless/Reversible	Low reaction time//Low precision/Small output force/UV is harmful	[75,76]
Bio		Biocompatibility	Low precision/Small output force	[77,78]
Hybrid		Reprogram/Multi-function/Cyclability	Multi-control and power apparatus	[67,79,80,81]

**Table 2:** Hydrogel-based soft robots.

Working Mechanism	The Form of Soft Robots	Reference
Thermal actuation	Finger	[91]
Skeletal muscle tissue recovery actuation	Bio-actuators	[102]
Light actuation (photothermal)	Earthworm	[74]
Electrically actuation (ion migration)	Gripper	[43,84]
Hydraulic actuation	Camouflage robots/Gripper	[64,96]
Pneumatic actuation	Bionic jellyfish	[109]
pH actuation	Drug delivery robot	[31,32]
Magnetic actuation	Actuators	[39]
Change the color according to the cell elongation/contraction	Soft structural color robotics	[110]
Change the color according to varied volume/thickness/temperature	Color sensor	[103,111]
The contact between IPTGRCV/GFP and RhamRCV/GFP with engineered bacteria resulted in fluorescence	Bio-sensors	[104]
Change the capacitance upon diverse temperature/pressure	Temperature/pressure sensors	[90]
The dynamic CNCs-Fe^3^⁺ coordination bonds change based on the strain	Strain sensors	[112]
Change the resistance by the varied strain	Strain sensors	[113,114]
Change the capacitance upon force	Touch sensor	[115]
Change electronic conductivity/fluorescence according to the AChE concentration	Electrochemical/fluorescence biosensor	[116]
Change the resistance by touch	Touch sensor	[117]
Change the voltage by touch	Touch sensor	[118]
Change the pH according to the reflected acoustic waves	Biochemical sensor	[119]
Change the transparency by different voltage	Organic liquid-crystal devices	[105]
Change the color under UV illumination at different times/temperatures/environments	Visual display	[106]
Change the color under the varied voltage	Electrochromic devices	[94]
Change the color/capacitance under the varied voltage	Electrochromic supercapacitor	[54]
The microstructure changing of conductive polymer hydrogels	Supercapacitors	[85]
The strong intermolecular interactions among GO and PANI, including electrostatic interaction, hydrogen bond effect, and π–π stacking.	Supercapacitors	[120]
The rheological properties of the electrode/electrolyte pastes are fine-tuned by varying the colloidal network structure.	Supercapacitors	[121]
Change the output force by the voltage input	Vibrotactile actuator	[87]
Change the output shape by the input voltage waveform	Haptic actuator	[122]
Harvest energy from the environment and convert it into electricity	Triboelectric Nanogenerators/Generators	[123,124]
The high stretchability/conductivity of the electronics that are made of Ag ink/thin elastomer/hydrogel layers)	Conductor	[86]
